# Interactive effect between sleep and exercise on depressive symptoms in Chinese adolescents

**DOI:** 10.3389/fpsyt.2023.1207243

**Published:** 2023-07-21

**Authors:** Shoukang Zou, Fang Deng, Wenli Tan, Yutong Fu, Hang Zhang, Hanmei Xu, Yuanmei Tao, Xian Tang, Xiaowei Tang, Ping Xiong, Huiping Huang, Ying Huang, Ling Li, Wenjuan Yang, Hongping Zeng, Gui Liu, Xiaosu Shen, Hongqin Zhao, Ying Chen, Kangling Yao, Jingyi Zhao, Wenwen Han, Jingmiao Zhou, Jianmin Hou, Shikun Peng, Yadan Wang, Yunzhen Yang, Yi Feng, Lin Chen, Xiting Yang, Shuangshuang Li, Xue Luo, Yan Wang, Li Yin

**Affiliations:** ^1^Mental Health Center, West China Hospital of Sichuan University, Chengdu, Sichuan, China; ^2^The Fourth People's Hospital of Chengdu, Chengdu, Sichuan, China; ^3^Chengdu Research Institute of Education Science, Chengdu, Sichuan, China; ^4^Center for Disease Control and Prevention, Chengdu, Sichuan, China; ^5^Chengdu Engineering Technical Vocational School, Chengdu, Sichuan, China; ^6^Sichuan Bright Foreign Language School, Emeishan, Sichuan, China; ^7^Chengdu Shishi Jincheng Foreign Language School, Chengdu, Sichuan, China; ^8^Sichuan Chengdu Zhonghe Vocational High School, Chengdu, Sichuan, China; ^9^Chengdu Eldo Primary School, Chengdu, Sichuan, China; ^10^Majiahe Primary School of Chengdu, Chengdu, Sichuan, China; ^11^Chengdu Huaxi Primary School, Chengdu, Sichuan, China; ^12^Shude Xiejin High School, Chengdu, Sichuan, China; ^13^Chengdu Wuhou Experimental Middle School Primary School, Chengdu, Sichuan, China; ^14^Yinxing Primary School, Chengdu, Sichuan, China; ^15^Southwest Jiaotong University Affiliated Middle School, Chengdu, Sichuan, China; ^16^Tianfu No. 4 High School, Chengdu, Sichuan, China; ^17^Chengdu Primary School Affiliated to Beijing International Studies University, Chengdu, Sichuan, China; ^18^Chengdu Shuangqing Primary School, Chengdu, Sichuan, China; ^19^Chengdu Shayan Primary School, Chengdu, Sichuan, China; ^20^Chengdu Xin Qiao Primary School, Chengdu, Sichuan, China; ^21^Institute for System Genetics, Frontiers Science Center for Disease-related Molecular Networks, Chengdu, Sichuan, China; ^22^Sichuan Clinical Medical Research Center for Mental Disorders, Chengdu, Sichuan, China

**Keywords:** sleep, exercise, interactive effect, adolescent, depressive symptoms

## Abstract

**Objectives:**

The study aimed to investigate the effects of sleep and exercise, individually and jointly, on depressive symptoms in Chinese adolescents.

**Methods:**

Cluster sampling was used to conduct a cross-sectional, electronic survey among 11,563 students from five primary and high schools in Sichuan Province in Western China. The questionnaire contained custom-designed items concerning sleep and exercise, while it used the Center for Epidemiologic Studies Depression Scale to assess depressive symptoms and the Core Self-Evaluations Scale to assess core self-evaluation. Data were analyzed using descriptive statistics and multivariate linear regression.

**Results:**

A total of 10,185 valid questionnaires were collected, corresponding to an effective response rate of 88.1%. Among the respondents in the final analysis, 5,555 (54.5%) were boys and 4,630 (45.5%) were girls, and the average age was 15.20 ± 1.72 years (range, 11–18 years). Only less than half of the respondents (4,914, 48.2%) reported insufficient sleep, while the remainder (5,271, 51.8%) had adequate sleep. Nearly one-quarter (2,250, 22.1%) reported insufficient exercise, while the remainder (7,935, 77.9%) reported adequate exercise. More than half of the respondents (5,681, 55.7%) were from vocational high school, 3,368 (33.1%) were from junior high school, 945 (9.3%) were from senior high school, and 191 (1.9%) were from primary school. The prevalence of depressive symptoms among all respondents was 29.5% (95% CI 28.7%−30.4%). When other variables were controlled, the depression score did not vary significantly with gender (*B* = −0.244, SE = 0.127, *P* = 0.054), but it decreased by 0.194 points per 1-year increase in age (*B* = −0.194, SE = 0.037, *P* < 0.001). Students getting adequate sleep had depression scores 2.614 points lower than those getting insufficient sleep (*B* = −2.614, SE = 0.577, *P* < 0.001), while students who engaged in adequate exercise had depression scores 1.779 points lower than those not exercising enough (*B* = −1.779, SE = 0.461, *P* < 0.001). The depression score decreased by 0.919 points per 1-point increase in the core self-evaluation score (*B* = −0.919, SE = 0.008, *P* < 0.001). In regression controlling for gender, age, and core self-evaluation, sleep and exercise were found to be related significantly to influence depressive symptoms (*B* = 0.821, SE = 0.315, *P* = 0.009).

**Conclusion:**

Adequate sleep and adequate exercise are individually associated with milder depressive symptoms in Chinese adolescents. Our results further highlight the need for researchers and clinicians to take into account not only the individual but also the joint effects of sleep and exercise on depression in adolescents when conducting research and designing interventions. If sleep or physical exercise has substantially reduced the risk of depressive symptoms, further reductions by improving sleep and exercise become difficult and may even have opposite effects.

## 1. Introduction

Depression often begins in adolescence, and a meta-analysis of 37 studies of high school students in China indicated a prevalence of 28% for depressive symptoms (1), while a meta-analysis of 72 studies of adolescents worldwide indicated a prevalence of 34%. The prevalence may be even higher among adolescents in the Middle East, Africa, and Asia (2). Thus, depression is a major disease, threatening the physical and mental health of adolescents. Identifying more influencing factors of depression may help to provide more options for the prevention and treatment of depression.

The National Sleep Foundation in the USA and the 24-Hour Movement Guidelines for Children and Youth in Canada recommend that children aged 5–13 years should sleep 9–11 h per night, while adolescents aged 14–17 years should sleep 8–10 h per night (3, 4). A survey on adolescent sleep in the USA found that only 26% of adolescents had adequate sleep (5). A lack of sleep affects the growth and development of adolescents and increases the risk of hypertension, diabetes, obesity, and other diseases (6, 7). It also increases the risk of depression and anxiety symptoms (8–10).

Regular, moderate physical exercise has been observed to reduce the risk of depression in adolescents and improve their mental health (11). The World Health Organization recommends that teenagers spend more than 1 h per day on average exercising at moderate intensity (12). In fact, a meta-analysis of prospective cohort studies found that physical exercise can reduce the incidence of depression among people of all ages (13). Among adults who did not exercise, those who completed half the recommended amount of exercise were at an 18% lower risk of depression, while those who completed the full amount were at a 25% lower risk (14).

Many studies have reported a correlation between core self-evaluation (CSE) and depression. CSE is an individual's basic evaluation of self-worth and ability, and it comprises mainly four traits: self-esteem, generalized self-efficacy, locus of control, and emotional stability (low neuroticism) (15–17). One study among Chinese university students found that fatalism and CSE mediated the relationship between stressful life events and depression (18). Another study reported that depression among Chinese adolescents correlated negatively with CSE and self-efficacy in expressing positive emotions and managing negative ones (19).

In addition to the effects of sleep and exercise individually on the risk of depression among adolescents, several studies have pointed to a potential interaction between them. For example, a 2-year longitudinal study of Estonian adolescent girls indicated an association between increased sleep disturbance and decreased physical activity with increased depressive symptoms (20). In addition, higher levels of depressive symptoms at the baseline in that study were associated with less physical activity and more sleep disturbance 2 years later. A systematic review study found that sleep deprivation contributed to the risk of depressive mood, while exercise reduced that risk (21). A study of high school students in Eastern China found that sleep duration and the number of days when respondents completed 60 min of physical exercise mediated the relationship between academic stress, anxiety, and depression (22). A study of Japanese high school students detected an interactive effect of sleep and exercise on depression (23).

This literature suggests that sleep and exercise act individually and jointly to influence the risk of depression among adolescents. However, large studies are needed to verify this hypothesis and begin to explore the causes. Therefore, we conducted a cross-sectional survey of more than 10,000 adolescents in Sichuan Province in Western China to explore relationships among sleep, exercise, and depressive symptoms.

## 2. Objectives and methods

### 2.1. Sample

Using PASS software, we calculated a minimal sample size of 8,165 assuming a prevalence of 30% for depressive symptoms among adolescents (2, 24, 25), tolerance error of 0.01, type I error of 0.05, and statistical power of 0.8. We increased the sample size by 10% to 9,072 in order to account for invalid questionnaires or lack of consent to participate. From October 2020 to March 2021, five primary and high schools in Chengdu (one junior high school, one primary school, and two vocational high schools) and Leshan (one high school containing a junior high school department and a senior high school department) were selected using cluster sampling. Teachers at each school sent electronic questionnaires to all students who are at least 11 years old. The final sample of 10,185 exceeded the minimal sample size, suggesting good statistical power.

### 2.2. Tools

Depressive symptoms were assessed using the Center for Epidemiologic Studies Depression Scale (CES-D), which contains 20 self-report items to which subjects respond using a 4-point scale from 0 to 3 based on the frequency of depression emotion or mood during the previous week. The CES-D can be divided into four factors: (1) physical symptoms, (2) negative emotion, (3) positive emotion, and (4) interpersonal relationships. Higher scores indicate higher levels of depression (26, 27). We defined a CES-D score of at least 20 as indicating the presence of depressive symptoms; this cutoff was found to balance sensitivity and specificity better in a systematic review study (28).

CSE was evaluated using the Core Self-Evaluations Scale (CSES), which contains 10 self-report items to which subjects respond on a 5-point scale from 1 to 5 according to how much they agree with a statement (1 = “completely disagree” and 5 = “completely agree”) (15, 29).

Data on sleep and exercise were obtained through a custom-designed set of questionnaires asking about the average number of hours of sleep per day, for which possible responses were “4 h or less,” “5 h,” “6 h,” “7 h,” “8 h,” “9 h,” “10 h,” “11 h,” or “12 h or more.” Subjects were also asked to report the average number of hours of medium- or high-intensity exercise in which they engaged daily, for which possible responses were “ < 1 h,” “1 h,” “2 h,” “3 h,” “4 h,” and “5 h or more.”

We defined adequate sleep as at least 9 h per day if the subject was 13 years old or younger or at least 8 h per day if the subject was at least 14 years old (3, 4). We defined adequate exercise as at least 1 h per day (12).

### 2.3. Research permission and informed consent

This study was approved by the Ethics Committee of West China Hospital, Sichuan University. The legal guardians (parents) of the minors were informed of the significance, confidentiality, and safety of the study through an electronic informed consent form, which they signed electronically. Only subjects whose legal guardians signed the informed consent were included in the analysis.

### 2.4. Statistical analysis

All data were analyzed using SPSS 23.0 software. Descriptive statistics were used to describe demographic information, depression scores, and proportions of students reporting sufficient sleep or sufficient exercise. Multivariate linear regression was used to identify factors influencing depression and to detect a potential joint influence of sleep and exercise on depression (30, 31).

## 3. Results

### 3.1. Characteristics of the sample

Of the 11,563 questionnaires distributed, 10,185 valid questionnaires were returned, corresponding to an effective response rate of 88.1%. Among those respondents, 5,555 (54.5%) were boys and 4,630 (45.5%) were girls; their average age was 15.20 ± 1.72 years (range, 11–18 years; Table 1). Only less than half of the respondents (4,914, 48.2%) reported insufficient sleep, while the remainder (5,271, 51.8%) had adequate sleep. Nearly one-quarter (2,250, 22.1%) reported insufficient exercise, while the remainder (7,935, 77.9%) reported adequate exercise. More than half of the respondents (5,681, 55.7%) were from vocational high school, 3,368 (33.1%) were from junior high school, 945 (9.3%) were from senior high school, and 191 (1.9%) were from primary school. The prevalence of depressive symptoms among all respondents was 29.5% (95% CI 28.7–30.4%), which had been reported in our previous article (32).

**Table 1:** Demographic information (number of students, %/*X* ± *S*).

**Items**	**Distribution**
**Gender**	
Male	5,555 (54.5%)
Female	4,630 (45.5%)
Age	15.20 ± 1.72 years
**Ethnic group**	
Han nationality	9,792 (96.2%)
Tibetan	178 (1.7%)
Yi nationality	112 (1.1%)
Other nationalities	103 (1.0%)
**Grade**	
Primary school	191 (1.9%)
Junior high school	3,368 (33.1%)
Senior high school	945 (9.3%)
Vocational high school	5,681 (55.7%)
**Sleep**	
Insufficient sleep	4,914 (48.2%)
Adequate sleep	5,271 (51.8%)
**Exercise**	
Insufficient exercise	2,250 (22.1%)
Adequate exercise	7,935 (77.9%)
CSES score	35.04 ± 7.81
CES-D score	15.95 ± 9.59
Physical symptoms	3.20 ± 3.20
Negative emotion	5.12 ± 5.05
Positive emotion	6.65 ± 3.33
Interpersonal relationships	0.99 ± 1.39

### 3.2. Factors influencing depressive symptoms

Multivariate linear regression was used to assess the effects of age, sex, CSE, sleep, and exercise on CES-D scores. In the case of sleep and exercise, we examined these factors individually and jointly. Before an interaction between sleep and exercise was specified, the inclusion of the five variables in the regression led to a significant model (*F* = 2,733.161, *P* < 0.001), which explained 57.3% of the observed variation in CES-D scores. No multicollinearity was observed among the included variables. Including interaction between sleep and exercise in the regression led to a model that was also significant (*F* = 2,280.072, *P* < 0.001) and that explained 57.3% of the observed variation in the CES-D scores.

When other variables were controlled, the depression score did not vary significantly with gender (*B* = −0.244, SE = 0.127, *P* = 0.054), but it decreased by 0.194 points per 1-year increase in age (*B* = −0.194, SE = 0.037, *P* < 0.001). Students getting adequate sleep had depression scores 2.614 points lower than those getting insufficient sleep (*B* = −2.614, SE = 0.577, *P* < 0.001), while students who engaged in adequate exercise had depression scores 1.779 points lower than those not exercising enough (*B* = −1.779, SE = 0.461, *P* < 0.001). The depression score decreased by 0.919 points per 1-point increase in the core self-evaluation score (*B* = −0.919, SE = 0.008, *P* < 0.001). In regression controlling for gender, age, and core self-evaluation, sleep and exercise were found to interact significantly to influence depressive symptoms (*B* = 0.821, SE = 0.315, *P* = 0.009) (Table 2).

**Table 2:** Multiple linear regression analysis of depressive symptoms-related factors.

**Independent variables**	***B*-value**	**SE**	***B*-value 95% CI**	***P*-value**
Constant	56.316	1.089	(54.181, 58.451)	< 0.001
Gender (male/female)	−0.244	0.127	(−0.493, −0.004)	0.054
Age	−0.194	0.037	(−0.267, −0.120)	< 0.001
Sleep	−2.614	0.577	(−3.746, −1.482)	< 0.001
Exercise	−1.779	0.461	(−2.683, −0.874)	< 0.001
CSES	−0.919	0.008	(−0.935, −0.903)	< 0.001
Sleep ^*^ exercise	0.821	0.315	(0.205, 1.438)	0.009

## 4. Discussion

In this study, 10,185 adolescents aged 11–18 years old from five primary and high schools in Chengdu and Leshan, China were investigated for depressive symptoms and influencing factors. Our data indicated a higher prevalence of depressive symptoms among students who lacked sleep or exercise. We also found that depressive symptoms correlate negatively with age and CSE.

The proportion of respondents in our study who reported insufficient sleep was 48.2%, which is similar to that reported among Saudi adolescents (33) but lower than that reported among USA and Chinese adolescents (34, 35). The proportion of respondents in our study who reported insufficient exercise was 22.1%, which is lower than that in studies in India and similar to that in the ABCD cohort study in the USA (36, 37), which may reflect that more than half of our respondents were in vocational high school, where academic demands are less than in other types of high school, leaving students more time and opportunities to engage in sports.

Our results are consistent with previous studies linking lower physical activity with a greater risk of depressive symptoms in the Avon Longitudinal Study of Parents And Children (ALSPAC) cohort from the University of Bristol (38), a meta-analysis of 23 randomized trials showing physical exercise to be effective for treating depression (39), and a longitudinal study in Europe showing that regular leisure-time exercise of any intensity reduced risk of depression during an 11-year follow-up (40). Our results are also consistent with a previous study linking adequate sleep to a lower risk of depressive symptoms (41, 42). Finally, our detection of an interactive effect of sleep and exercise on depression among Chinese adolescents mirrors the results from a similar study in Japan (23). These observations suggest the robustness of the effects of sleep and exercise, individually and jointly, on the risk of depression among adolescents.

Interestingly, the interactive effect of sleep and exercise in our study was in the opposite direction as their individual effects on depressive symptoms. The Japanese study reported a similar finding (23). This may reflect an overlap in how sleep and exercise influence such symptoms, such that if sleep or physical exercise has substantially reduced the risk of depressive symptoms, further reductions by improving sleep and exercise become difficult. The finding may also reflect the influence of other factors that we did not take into account, such as negative life events, school bullying, family history of mental illness, or personality traits.

Since this was a cross-sectional study, we could not make a causal inference. We did not objectively measure sleep or exercise but relied on self-report, increasing the risk of memory and reporting bias. It may be more objective to survey subjects using the Pittsburgh Sleep Quality Index or to monitor their sleep or exercise directly using, for example, a wearable device (38, 43, 44). We did not collect data about the history of mental illness, which may have contributed to depressive symptoms. All subjects in this study were adolescents from Sichuan province, so whether our results can be generalized to other ethnic and age groups is unknown. We did not rigorously determine whether students had depression or not, but only examined their depressive symptoms, which means that our findings should be confirmed in adolescents diagnosed with depression. Indeed, these limitations mean that our results should be interpreted with caution.

Despite these limitations, our study, together with an increasing number of other studies, suggests that ensuring adequate sleep duration and increasing physical activity in adolescents can mitigate and even prevent depressive symptoms. Our results further highlight the need for researchers and clinicians to take into account not only the individual but also the joint effects of sleep and exercise on depression in adolescents when Conducting research and designing interventions. If sleep or physical exercise has substantially reduced the risk of depressive symptoms, further reductions by improving sleep and exercise become difficult and may even have opposite effects. In future, we hope to conduct a large, longitudinal study and analyze more factors related to depressive symptoms in order to explore how sleep and exercise affect such symptoms.

## Data availability statement

The original contributions presented in the study are included in the article/supplementary material, further inquiries can be directed to the corresponding author.

## Ethics statement

The studies involving human participants were reviewed and approved by the Ethics Committee of West China Hospital, Sichuan University. Written informed consent to participate in this study was provided by the participants' legal guardian/next of kin.

## Author contributions

All authors listed have made a substantial, direct, and intellectual contribution to the work and approved it for publication.
